# Genome-wide analysis and functional characterization of the *SUT* gene family associated with stress tolerance in *Glycine max*

**DOI:** 10.3389/fpls.2026.1717720

**Published:** 2026-02-11

**Authors:** Tianyi Cui, Rong He, Pengwei Wang, Zhen Zhu, Han Xing, Jinming Zhao, Na Guo

**Affiliations:** 1 Key Laboratory of Biology and Genetics and Breeding for Soybean, Ministry of Agriculture, Nanjing, China; 2 Zhongshan Biological Breeding Laboratory (ZSBBL), National Innovation Platform for Soybean Breeding and Industry-Education Integration, Nanjing, China; 3 State Key Laboratory of Crop Genetics & Germplasm Enhancement and Utilization, Nanjing Agricultural University, Nanjing, China; 4 Jiangsu Key Laboratory of Soybean Biotechnology and Intelligent Breeding, College of Agriculture, Nanjing Agricultural University, Nanjing, China

**Keywords:** abiotic stress response, *GmSUT* protein interaction network, phytohormone response, soybean, sucrose transporter gene family

## Abstract

**Background:**

Soybean (*Glycine max*), is a globally important oilseed crop whose yield and quality are severely constrained by environmental stress. The Sucrose Transporter (*SUT*) gene family plays a crucial role in sucrose transport, plant growth, and stress adaptation. However, comprehensive identification and functional characterization of *SUT* family members in soybean remain largely incomplete.

**Results:**

In this study, a total of 12 non-redundant *GmSUT* genes were identified in soybean. The encoded proteins have predicted molecular weights ranging from 11.80 to 65.88 kDa and theoretical isoelectric points (pI) between 5.73 and 9.44. These genes were classified into three subfamilies (*SUTI*, *SUTIIa* and *SUTIV*) by phylogenetic analysis, with SUTI being the largest group. Gene structure and conserved motif analyses showed that motif composition was largely uniform within each subfamily, except for *GmSUT4.1*, which retained only two motifs. Chromosomal mapping revealed an uneven distribution across seven chromosomes, with Chr16 harboring four SUTI members. Collinearity analysis indicated a closer evolutionary relationship between soybean and *Glycine soja* than with *Arabidopsis thaliana* or *Medicago truncatula*. Cis −regulatory element analysis identified abundant stress- and hormone-responsive motifs (e.g., ABRE, MeJA−responsive elements), with 83% of promoters containing ABA-responsive elements. Moreover, the transcriptional levels of the *GmSUT* genes were significantly induced under various abiotic stresses (salt, drought, cold and alkaline) and phytohormone treatments (ABA, and MeJA), demonstrating that multiple *GmSUT* genes play critical roles in soybean stress adaptation.

**Conclusions:**

This study provides a comprehensive identification and characterization of the *SUT* gene family in soybean (*Glycine max*), revealing 12 *GmSUT* genes grouped into three subfamilies (SUTI, SUTIIa, SUTIIV). Expression profiling demonstrated that multiple *GmSUT* members are rapidly upregulated under stress treatment, underscoring their essential functions in sucrose distribution and stress adaptation. These findings offer valuable insights into the regulatory mechanisms of the *GmSUT* family and suggest candidate genetic targets for enhancing stress tolerance in soybean.

## Introduction

Soybean (*Glycine max*) is a globally vital crop, serving as a major source of edible oil and plant-based protein. However, its productivity is significantly constrained by abiotic stresses, including drought, salinity, extreme temperatures, and soil alkalinity, which collectively cause annual yield losses estimated between 15% and 20% worldwide (Dong et al., 2021; Hu et al., 2022; Saleem et al., 2022; Shao et al., 2023). Sugars, principally derived from photosynthesis, are indispensable not only as energy substrates but also as key signaling molecules that orchestrate plant development and stress adaptation (Paul et al., 2001). Among them, sucrose acts as the primary form of translocated carbon in higher plants and plays a central role in coordinating source–sink allocation between photosynthetic tissues and heterotrophic organs such as roots, developing seeds, and storage tissues. Consequently, precisely regulated sucrose transport is crucial for sustaining growth, ensuring yield stability, and maintaining stress resilience, particularly under adverse environmental conditions (Wen et al., 2022; Fan et al., 2023).

Sucrose Transporters (*SUT*s) constitute a gene family responsible for the transmembrane movement of sucrose. This family is divided into three subfamilies—SUTI, SUTII, and SUTIV, with SUTII further divided into SUTIIa and SUTIIb. SUTI genes are predominantly found in dicots and play critical roles in sucrose translocation between source and sink tissues. In contrast, SUTIIb appears to be specific to monocots. SUTIIa and SUTIV are present in both monocots and dicots, suggesting conserved yet functionally distinct roles (Li et al., 2014; Yadav et al., 2022).

Emerging research highlights the diverse functions of *SUTs* in plant growth, development, and stress adaptation. In rice, for instance, suppression of *OsSUT1* has been shown to improve salt tolerance by maintaining sucrose levels in roots and enhancing photosynthetic efficiency (Siahpoosh et al., 2012). Under drought stress, *OsSUT1* expression is upregulated to promote sucrose transport to roots, whereas *OsSUT4* expression is downregulated, reflecting functional specialization within the SUT family (Xu et al., 2018). Additionally, during grain filling, heat stress leads to reduced expression of *OsSUT1* and starch synthesis genes, resulting in premature maturity and chalky grains (Miyazaki et al., 2013; Phan et al., 2013; Xu et al., 2018). In chili peppers, the expression of *CaSUT* genes likewise varies in response to heat, cold, and salt stress, further underscoring the involvement of *SUTs* in abiotic stress adaptation (Chen et al., 2022).

The expression of sucrose transporters is modulated by phytohormone signaling pathways, which integrate developmental and environmental stress cues. Abscisic acid (ABA), a central hormone in abiotic stress responses, regulates *SUT* genes across multiple species. For instance, in Arabidopsis, *AtSUC2* and *AtSUC4* are required for stress tolerance via an ABA-dependent pathway (Gong et al., 2015). Beyond ABA, other phytohormones such as auxin also influence sucrose transport. In rice, for example, auxin directly regulates *OsSUT1* to fine-tune carbohydrate partitioning between source and sink tissues and coordinate reproductive development (Zhao et al., 2022). In soybean, drought and associated hormonal changes coincide with altered expression of sugar transporters and phloem-related genes, highlighting functional coordination between hormone signaling and sucrose transport under stress (Hu et al., 2022). Collectively, these studies demonstrate that *SUT*s are embedded within hormone-responsive regulatory networks essential for stress adaptation.

In a previous study, Guo et al. (2024) conducted a genome-wide identification of all sugar transporter families in soybean. Their work characterized the tissue-specific expression profiles of these transporters and analyzed their transcriptional responses to abiotic stresses, including salinity, drought, and cold, by mining public transcriptomic datasets. Despite these advances, the responses of sucrose transporters (*SUT*s) to phytohormones remain largely unexplored. To address this knowledge gap, we performed a comprehensive genome-wide identification of the sucrose transporter (*SUT*) gene family in soybean. Our analysis focused on elucidating their evolutionary relationships, gene structures, and expression patterns under various abiotic and hormonal stresses. Furthermore, we evaluated the potential of these *SUT* genes as strategic targets for enhancing stress tolerance in soybean breeding programs. Collectively, our findings provide a valuable resource for subsequent functional characterization of sucrose transporters. They also offer a foundational framework for leveraging these genes in the development of soybean cultivars with improved seed yield and resilience to environmental stresses.

## Results

### Identification and characterization of the *SUT* gene family in *Glycine max*

A total of 12 non-redundant *GmSUT* genes were manually selected based on the results from the two identification methods. The members of this gene family were named based on their respective subfamilies as *GmSUT1.1*, *GmSUT1.2*, *GmSUT1.3*, *GmSUT1.4*, *GmSUT1.5*, *GmSUT1.6*, *GmSUT1.7*, *GmSUT2.1*, *GmSUT2.2*, *GmSUT4.1*, *GmSUT4.2* and *GmSUT4.3*. The physicochemical properties of the *GmSUT* genes are summarized in **Supplementary Table S1**, including amino acid length, molecular weight, theoretical pI, instability index, aliphatic index, and the grand average of hydropathicity. The molecular weights of the GmSUT proteins ranged from 11.80 kDa to 65.88 kDa, with the highest pI value being 9.44 (*GmSUT4.2*) and the lowest pI value being 5.73 (*GmSUT2.2*). They encode proteins with a size range of 105 to 615 aa. Subcellular localization prediction suggested that most GmSUT proteins are localized to the plasma membrane, with several also predicted to localize to the cytoplasm. Notably, *GmSUT4.2* and *GmSUT4.3* were predicted to localize to the mitochondrion and chloroplast, respectively (**Supplementary Table S2**).

### Phylogenetic analysis of GmSUT proteins

**Figure 1:**
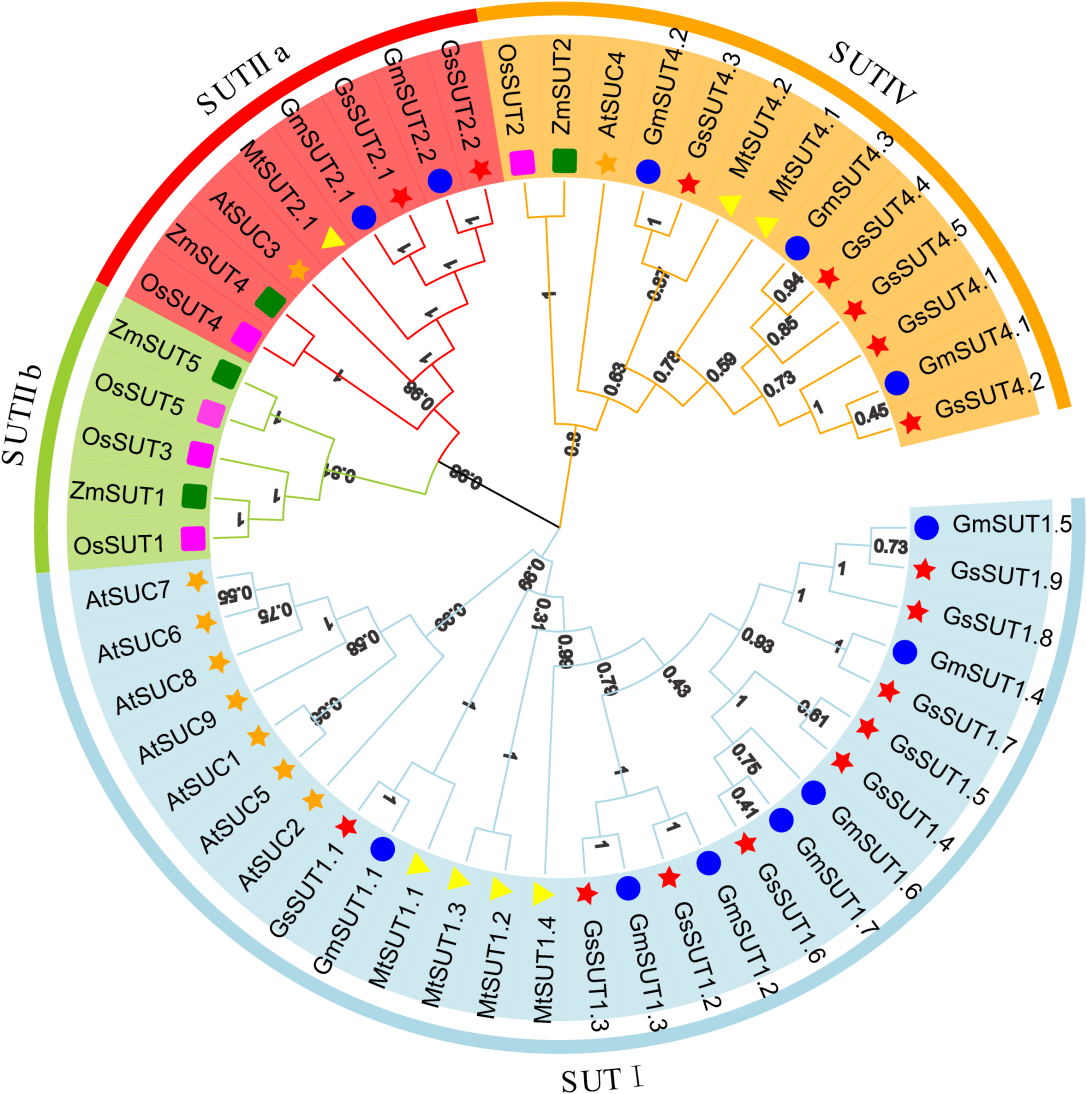
Phylogenetic tree of SUT proteins from Arabidopsis, rice, soybean, maize, wild soybean and *Medicago truncatula*. The phylogenetic tree was assembled using a dataset of 53 protein sequences, including 9 from *AtSUC*s, 5 from *OsSUT*s, 4 from *ZmSUT*s, 7 from *MtSUT*s, 16 from *GsSUT*s, and 12 from *GmSUT*s, and these sequences were grouped into four distinct subfamilies. SUT genes from Arabidopsis, rice, soybean, maize, wild soybean and *Medicago truncatula* are denoted by orange star, magenta square, blue circle, green square, red star and yellow triangle, respectively. Details of the *SUT* genes from six species are listed in **Supplementary Table S3**. The tree was inferred in MEGA11 using the maximum-likelihood (ML) method and visualized in TBtools.

To better understand the evolutionary interrelatedness among these *GmSUT* members, we constructed a maximum-likelihood phylogenetic tree in MEGA11 using amino acid sequences of SUTs from six species (*Glycine max*, *Glycine soja*, *Oryza sativa*, *Zea mays*, *Arabidopsis thaliana*, and *Medicago truncatula*), and visualized the tree in TBtools (Chen et al., 2023) (**Figure 1**; **Supplementary Table S3**). The 12 *SUT* genes in *Glycine max* were unevenly distributed among subfamilies. The SUT gene family members of *Glycine soja* were obtained by the same method as that used for the previously identified soybean members, and were named according to the same rule. Based on previously reported subfamily classifications of SUT genes in *A. thaliana*, *O. sativa*, *M. truncatula*, and *Z. mays* (Sun et al., 2022; Usha et al., 2015; Ibraheem et al., 2010; Doidy et al., 2012), we divided *SUT* genes into four subfamilies: SUTI, SUTIIa, SUTIIb, and SUTIV. The SUTI subfamily was the largest subfamily with 7 GmSUT members, followed by the SUTIIa and SUTIV subfamilies with 2 and 3 members respectively, and the SUTIIb subfamily contains genes only from monocotyledonous plants. Members of the *SUT* family shared similar motif composition and distribution. In addition, the position and order of the motifs were similar within the *SUT* family.

### Gene structure, conserved motif, and multiple alignment analysis

**Figure 2:**
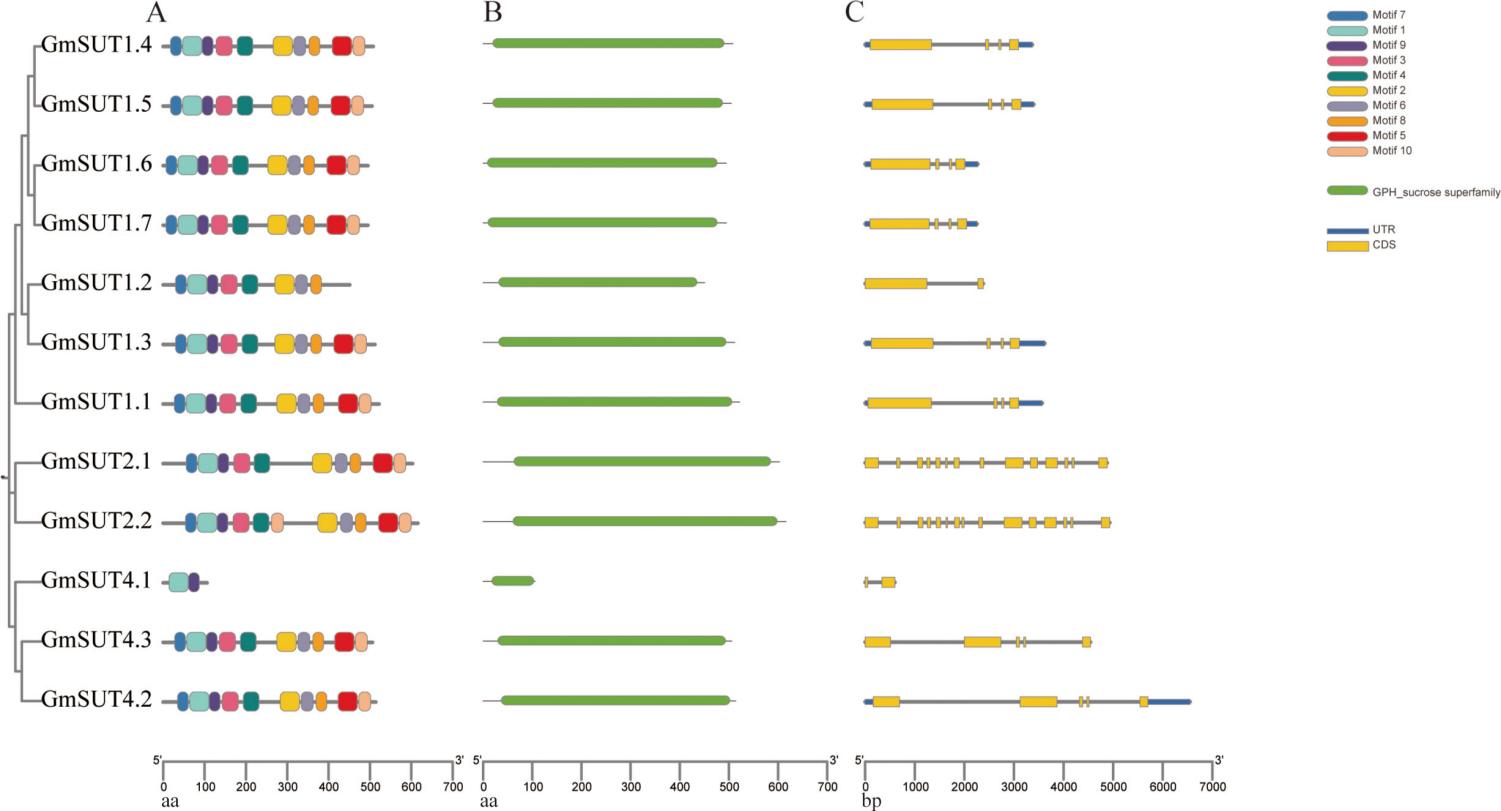
Analysis of the motif and gene structure of the SUT gene family in *Glycine max*. **(A)** Conserved motifs of *SUT* genes in *Glycine max*. Distribution of the 10 conserved motifs in the *GmSUT* genes following analysis by MEME tool. The different-colored boxes represent different motifs and their position in each protein sequence of SUT. **(B)** Domain analysis of SUT proteins in *Glycine max*. **(C)** Gene structure of *SUT* genes in *Glycine max*. Exons are indicated by yellow rectangles. Gray lines connecting two exons represent introns.

To understand the gene structure of the *SUT* genes of *Glycine max*, MEME tool was used to analyze 12 SUT gene family members of *Glycine max*, and TBtools was used to visualize the conserved motif of *GmSUT* genes (**Figure 2A**). A total of 10 conserved motifs were identified to characterize shared motifs among SUT proteins (**Supplementary Table S4**). The results showed that the number of conserved motifs in each protein ranged from 2 to 10, and all the conserved motifs were uniformly present in the protein sequences. For example, Motif 7, Motif 1, Motif 9, Motif 3 and Motif 4 exist in the N-terminal domain. Moreover, Motif 2, Motif 6, Motif 8, Motif 5, and Motif 10 existed in the C-terminal domain. Among all members, except for *GmSUT4.1*, all members have at least eight motifs, and the distribution is uniform, while *GmSUT4.1* has only two motifs, Motif 1 and Motif 9.

Domain analysis showed that *SUT* domains were uniformly present in the protein sequences (**Figure 2B**). Multiple sequence alignment further confirmed high sequence similarity among *GmSUT* family members, with conserved regions predominantly distributed in the functional domains (**Supplementary Figure S1**), supporting their evolutionary conservation and potential functional redundancy. All members contain the conserved domain of the GPH-sucrose superfamily. In addition, the conserved domain of GmSUT4.2 belongs to MFS-1 domain (PF07690) and the other members belong to MFS-2 domain (PF13347). To understand the gene structure of the SUT genes of *Glycine max*, intron-exon structure analysis was performed (**Figure 2C**). The results showed that all members of *GmSUT* genes contain introns; most members of the SUTI subfamily contain three introns except for *GmSUT1.2*, two members of the SUTIIa subfamily have 13 introns, and two of the three members of the SUTIV subfamily have four introns. In addition, the distribution of introns and exons in the same subfamily is similar, which may be related to functional differentiation of gene family. Although *GmSUT4.1* (the third member of the SUTIV subfamily) contains only one intron, is short in length, and harbors only Motif 1 and Motif 9, its conserved domain and gene structure are similar to other SUTIV subfamily members.

### Chromosomal localization, gene duplication, and collinearity analysis of *GmSUTs*

**Figure 3:**
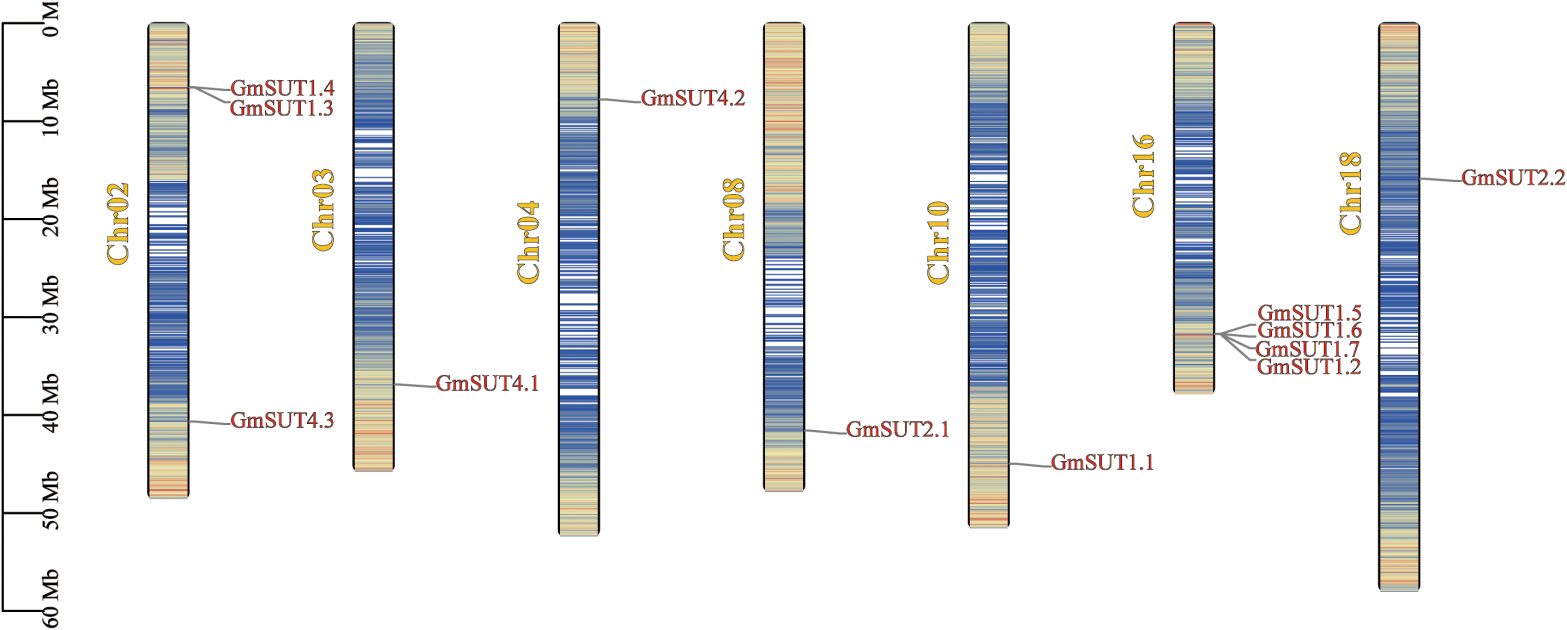
Chromosomal location of *SUT* genes in *Glycine max*. The 12 *SUT* genes are widely mapped to 7 chromosomes of *Glycine max*. The lines in the chromosome represent the density of the chromosome. Members of the *GmSUT* gene family are located on Chr2, Chr3, Chr4, Chr8, Chr10, Chr16, and Chr18.

The chromosomal localization of *SUT* genes was mapped based on the physical location of the genes in the *Glycine max* genome (**Figure 3**). The results showed that 12 *GmSUT* genes were unevenly distributed on 7 chromosomes. Most of these *GmSUT* genes are distributed on Chr02 and Chr16, with 3 and 4 genes. There is one member on each of the five chromosomes Chr03, Chr04, Chr08, Chr10 and Chr18. As shown in **Figure 3**, Chr16 contains four members of the SUTI subfamily of the *SUT* gene family. The remaining three members of SUTI are located in Chr02 and Chr10; two members of SUTIIa are located in Chr08 and Chr18; and three members of SUTIV are located in Chr02, Chr03, and Chr04.

Analysis of chromosomal localization revealed the presence of tandem duplications on Chr02, Chr08, Chr10 and Chr18, and a total of 6 tandem repeat genes were found (**Supplementary Table S5**). All Ka/Ks ratios for duplicated gene pairs were smaller than 0.5, indicating that these genes were subjected to purifying selection. Furthermore, a total of 10 paralogues were identified in *Glycine max SUT* gene family. All paralogues exhibited a Ka/Ks ratio of less than 0.5. Both segmental and tandem duplications have contributed to the expansion and diversification of the *GmSUT* gene family. Segmental duplications, such as between *GmSUT1.1/GmSUT1.3* and *GmSUT1.4/GmSUT1.5*, with Ka/Ks ratios of 0.18 and 0.14 (**Supplementary Table S5**), indicate purifying selection, likely reflecting retention after whole genome duplication (WGD) or ancient duplications, which drive gene family expansion in plants (Büttner, 2007; Zhang et al., 2021). Tandem duplications, like between *GmSUT2.1/GmSUT2.2* (Ka/Ks=0.29), enable functional diversification, allowing gene copies to evolve specialized roles in stress adaptation. These duplication patterns illustrate how WGD/segmental retention and tandem expansion shape *GmSUT* diversity, with some duplicates maintaining core functions and others diverging to support stress tolerance in soybean.

**Figure 4:**
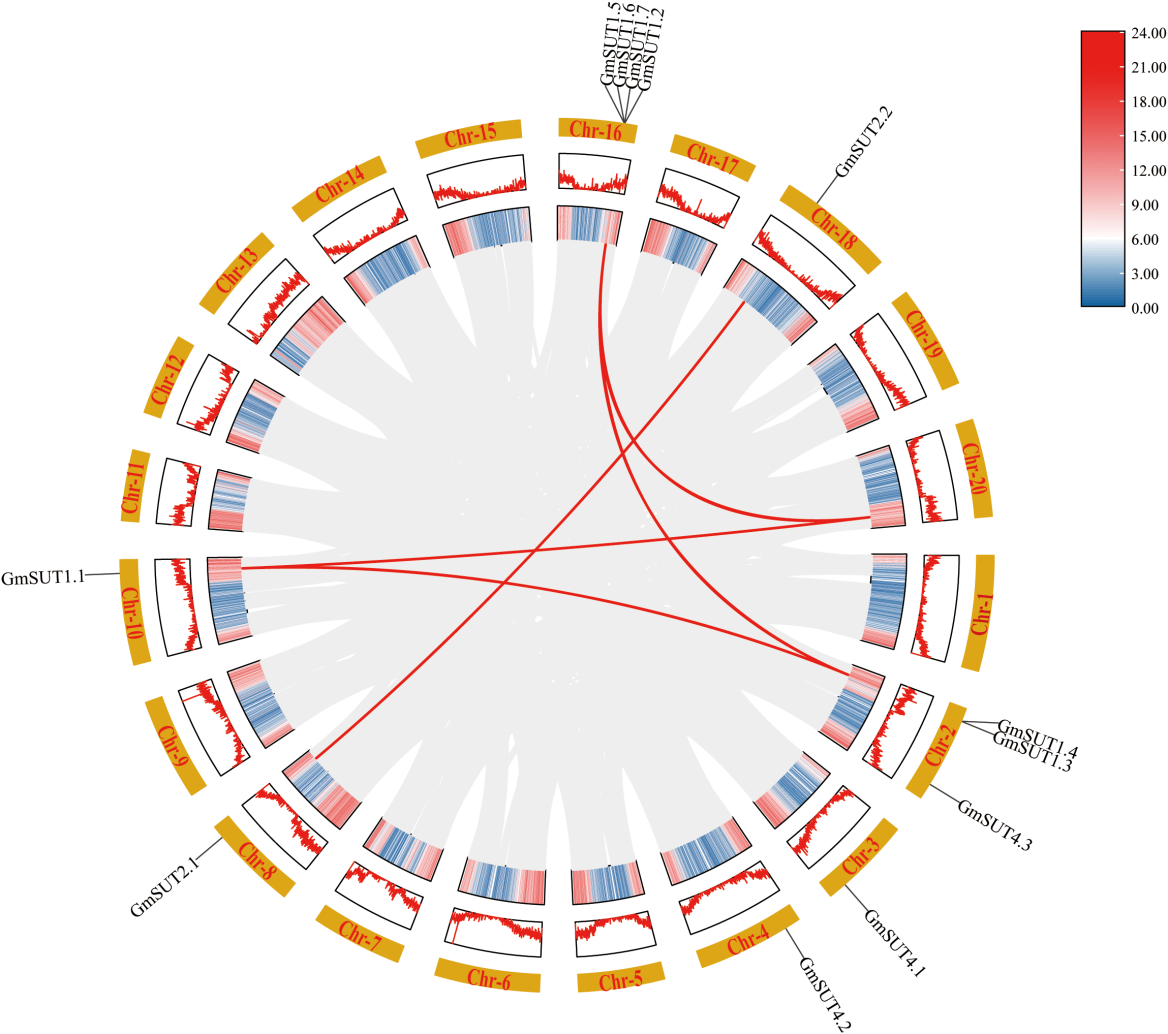
Distribution of *GmSUT*s segment duplication gene pairs on soybean chromosomes. The chromosome number is indicated on the inner side of each chromosome. The heatmap in the inner circle represents the gene density on the chromosome.

**Figure 5:**
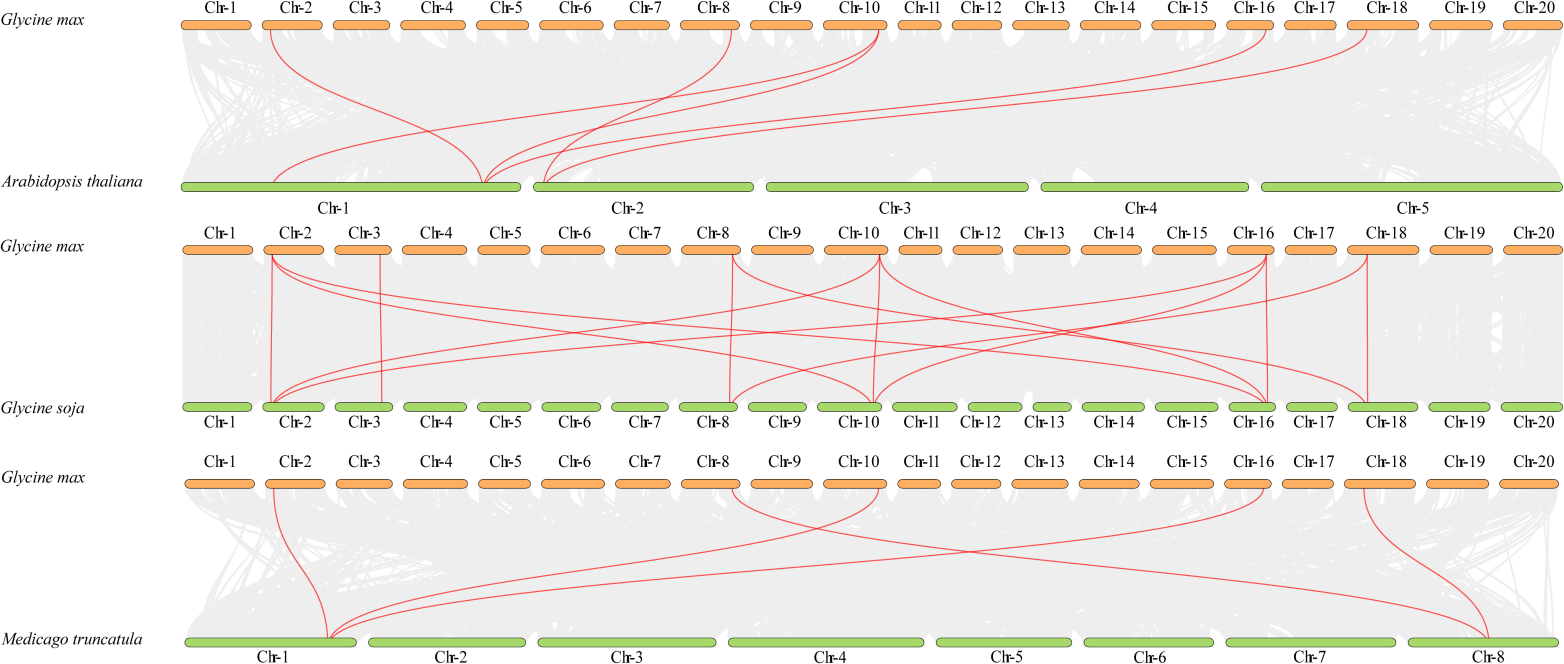
Collinearity analysis of *SUT* genes between *Glycine max* and three related species. The figure illustrates the syntenic relationships of *SUT* genes between *Glycine max* and three other species (*Arabidopsis thaliana*, *Glycine soja*, and *Medicago truncatula*). Red lines represent the collinearity pairs of *SUT* genes between *Glycine max* and the other species, while gray lines represent the collinearity pairs between other species. “Chr” is the abbreviation for chromosome. The chromosomes of *Glycine max* are depicted as orange rectangular blocks, and the chromosomes of the compared species are shown as green rectangular blocks. The names of the chromosomes are labeled within the corresponding colored blocks. The figure highlights the evolutionary conservation and divergence of the *SUT* gene family across these species.

Using MCScanX, we identified three collinear gene pairs among the 12 *GmSUT* genes (**Figure 4**), including one pair in the SUTI subfamily and one pair in the SUTIIa subfamily. Numerous collinear blocks were detected among *A. thaliana*, *G. soja*, *M. truncatula*, and *G. max* (**Figure 5**). In total, 6, 14, and 5 *GmSUT* genes showed synteny with genes from *A. thaliana*, *G. soja*, and *M. truncatula*, respectively.

### Identification and distribution of Cis-regulatory elements in *GmSUT* promoter

**Figure 6:**
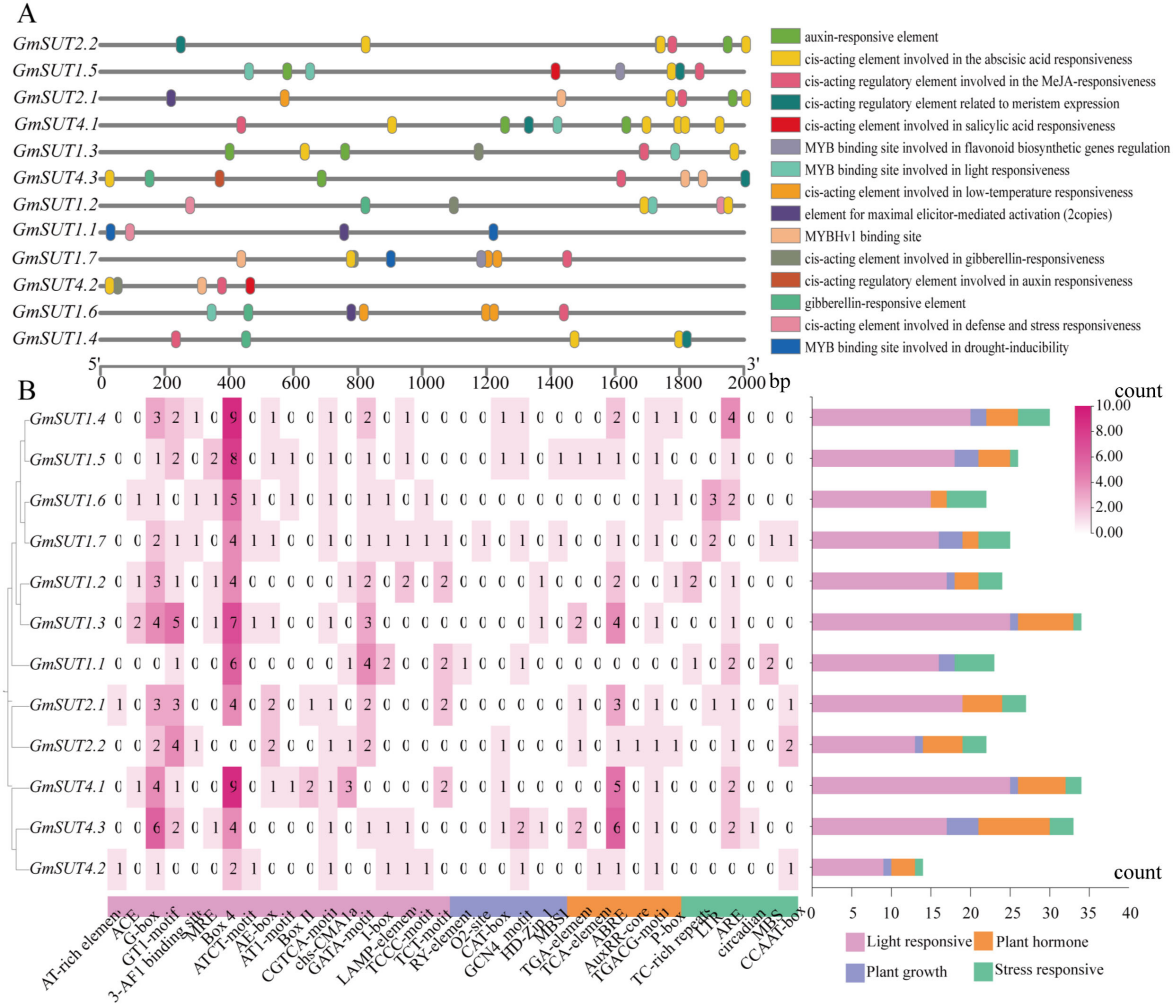
Analysis of cis-acting elements in the promoter region (2000 bp) of *GmSUT* genes. **(A)** The distribution of cis-acting elements related to abiotic stress in *GmSUT* genes. **(B)** Cis-acting elements analysis of *GmSUT* genes in promoter region of *Glycine max*. Left panel: Number of each cis-acting element in the promoter region (2000 bp) of *GmSUT* genes. Right panel: Statistics for the total number of *GmSUT* genes.

The upstream 2000 bp promoter region of 12 *GmSUT* genes was analyzed, and 331 elements were obtained, including light, growth development, hormone, and stress response elements. The members of the gene family contain all types of cis-acting elements; only *GmSUT1.1* does not contain plant hormone-responsive elements, as shown in **Figure 6**. The number of light-response elements was the largest, accounting for 84% of all elements. The hormone-related cis-acting regulatory elements in the *GmSUT* gene family promoters included abscisic acid-responsive element (ABRE), MeJA-responsive element (CGTCA-motif and TGACG-motif), gibberellin-responsive element (GARE-motif), salicylic acid-responsive element (TCA-element), and auxin-responsive element (AuxRR-core). Analysis revealed that 83% of *GmSUT* promoters (10 out of 12 genes) contained ABRE elements. The MeJA-responsive elements were also prevalent, with 75% of *GmSUT* promoters (9 out of 12) harboring both CGTCA-motif and TGACG-motif. Additionally, gibberellin-responsive element, auxin-responsive element, and salicylic acid-related elements were identified in 4, 3, and 3 *GmSUT* promoters, respectively.

Stress-responsive cis-elements such as ARE, LTR, MBS, TC-rich repeats, and GC-motif were detected. Notably, 42% of *GmSUT* promoters (5 out of 12) contained low-temperature responsive elements (LTR). For plant growth and development, 5 elements linked to processes like metabolic regulation were identified, accounting for 69% of developmental elements (5 out of 6). The flavonoid biosynthesis-related MBS element was uniquely present in *GmSUT1.1* and *GmSUT1.7*, while *GmSUT1.5* and *GmSUT1.7* contain MBSI, suggesting specialized regulatory functions in *GmSUT* family.

### Spatial and temporal expression pattern analysis of 12 *GmSUT* genes

**Figure 7:**
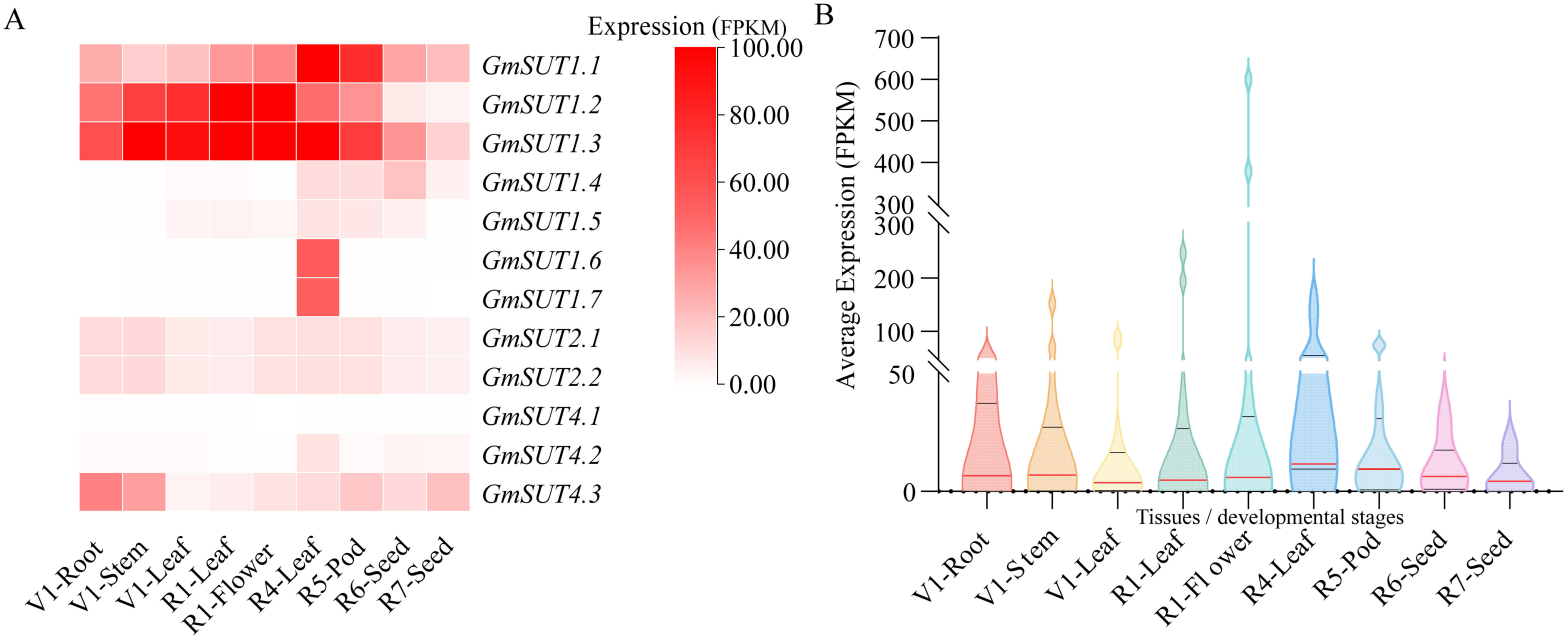
Spatiotemporal expression patterns of 12 *GmSUT* genes in *Glycine max*. **(A)** Hierarchical clustering heatmap of *GmSUT*s across 9 soybean tissues, with genes clustered into three expression groups. **(B)** Violin plot of *GmSUT* expression distribution in different tissues, showing R1-Flower has the highest median expression level. Data are FPKM-normalized and retrieved from the SoyMD database.

To characterize the spatiotemporal expression landscape of the *GmSUT* gene family, we analyzed FPKM-normalized transcriptome data for 12 *GmSUT* genes (*GmSUT1.1*–*GmSUT4.3*) across nine tissues representing key developmental stages (V1-Root, V1-Stem, V1-Leaf, R1-Leaf, R1-Flower, R4-Leaf, R5-Pod, R6-Seed, and R7-Seed) from 26 soybean varieties (**Supplementary Table S6**; **Figure 7**). Hierarchical clustering of the heatmap (**Figure 7A**) revealed three major expression patterns: (i) a high-expression cluster dominated by *GmSUT1.2* and *GmSUT1.3*, which showed strong enrichment in R1-stage flowers; (ii) a moderate and relatively stable cluster represented by *GmSUT1.1*, with preferential expression in R4 leaves and R5 pods; and (iii) a low-expression cluster including *GmSUT1.6*, *GmSUT1.7*, and *GmSUT4.1*, which displayed weak or near-silent expression across most tissues with only slight increases in R4 leaves.

The tissue-level distribution was further summarized by the violin plot (**Figure 7B**), which showed the highest overall expression in R1 flowers, followed by moderate expression in R4 leaves, whereas V1 roots, V1 stems, and R7 seeds exhibited the lowest expression. Collectively, these results indicate that *GmSUT1.2* and *GmSUT1.3* are the most prominent members during flowering, while a subset of *GmSUT*s is preferentially expressed in leaf and pod tissues, supporting functional differentiation of the family in development-associated sucrose allocation.

### Expression analysis of the *GmSUT* in response to abiotic stresses

**Figure 8:**
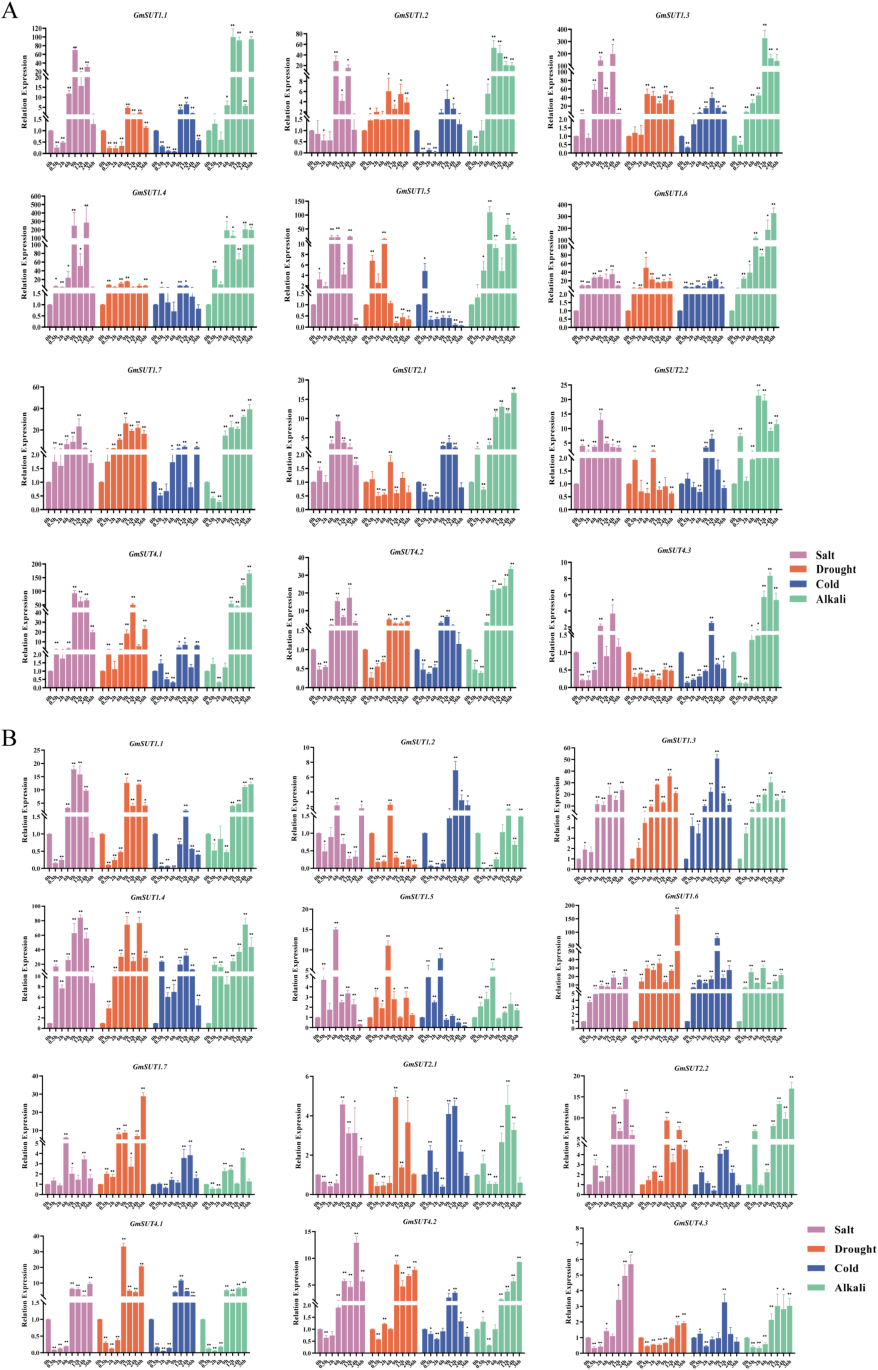
Expression analysis of 12 *GmSUT* genes following salt, drought, cold and alkali treatments by qRT-PCR. **(A)** The expression of *GmSUT*s in the root part. **(B)** The expression of *GmSUT*s in the shoot part. The Y-axis and X-axis indicate relative expression levels and the time courses of stress treatments, respectively. Statistical significance was performed using a paired Student’s t test. Mean values and standard deviations (SDs) were obtained from three biological and three technical replicates, and significant differences relative to controls were indicated at *P ≤ 0.05 and **P ≤ 0.01. The error bars indicate standard deviation.

The expression patterns of *GmSUT* gene family members in soybean roots and shoots were analyzed under salt, rapid drought, cold, and alkali stresses (**Figures 8A, B**).In roots, 8 out of 12 *GmSUT* members were upregulated under salt stress, peaking between 9 h and 24 h, while *GmSUT1.1*, *GmSUT1.2*, *GmSUT4.2*, and *GmSUT4.3* exhibited a “decrease-then-increase” pattern, also peaking at 9–24 h. Under rapid drought, only *GmSUT2.1*, *GmSUT2.2*, and *GmSUT4.3* were downregulated; the remaining members were largely upregulated with peaks at 9–12 h. Among them, *GmSUT1.1* and *GmSUT4.2* initially increased (0–6 h) before declining, whereas *GmSUT1.5* decreased initially (0–6 h) and subsequently rose, though its final expression remained below untreated levels. Cold stress induced a “decrease-then-increase” response in 9 of the 12 members, with peaks observed at 12–24 h; *GmSUT1.4* and *GmSUT1.6* were upregulated, while *GmSUT1.5* was inhibited. Under alkali stress, most members were upregulated except for the three SUTIV subfamily genes and *GmSUT1.7*, which displayed a “decrease-then-increase” pattern peaking at 36 h.In shoots, salt stress upregulated 6 of the 12 *GmSUT* members; three SUTIV genes and *GmSUT1.1* showed a “decrease-then-increase” trend, *GmSUT1.5* and *GmSUT1.7* exhibited an “increase-then-decrease” pattern, and *GmSUT1.2* was downregulated. Drought stress elicited expression profiles similar to those under salt stress, except that *GmSUT1.7* was upregulated. During cold stress, 8 of the 12 members first decreased (reaching their lowest levels at 6–9 h) and then increased; *GmSUT1.3*, *GmSUT1.4*, and *GmSUT1.6* were upregulated, while *GmSUT1.5* followed an “increase-then-decrease” trajectory. Under alkali stress, 6 members displayed a “decrease-then-increase” response with the lowest expression around 2 h; *GmSUT1.3*, *GmSUT1.4*, *GmSUT1.6*, and two SUTIIa members were upregulated, whereas *GmSUT1.5* peaked at approximately 6 h before declining.

All *GmSUT* genes responded differentially to the four stress treatments. Members of the SUTIIa and SUTIV subfamilies showed broadly similar expression trends and were mostly upregulated to varying extents. Only *GmSUT1.2* (in shoots under salt/drought) and *GmSUT1.5* (in roots under cold) were consistently downregulated.

### Expression analysis of the *GmSUT* in response to phytohormones

Phytohormones are integral to abiotic stress responses, playing pivotal roles in mediating plant adaptation to diverse adverse environmental conditions (Waadt et al., 2022). To investigate the hormone-responsive profiles of *GmSUT* genes, we examined their expression patterns in soybean shoots and roots following treatments with ABA and MeJA.

**Figure 9:**
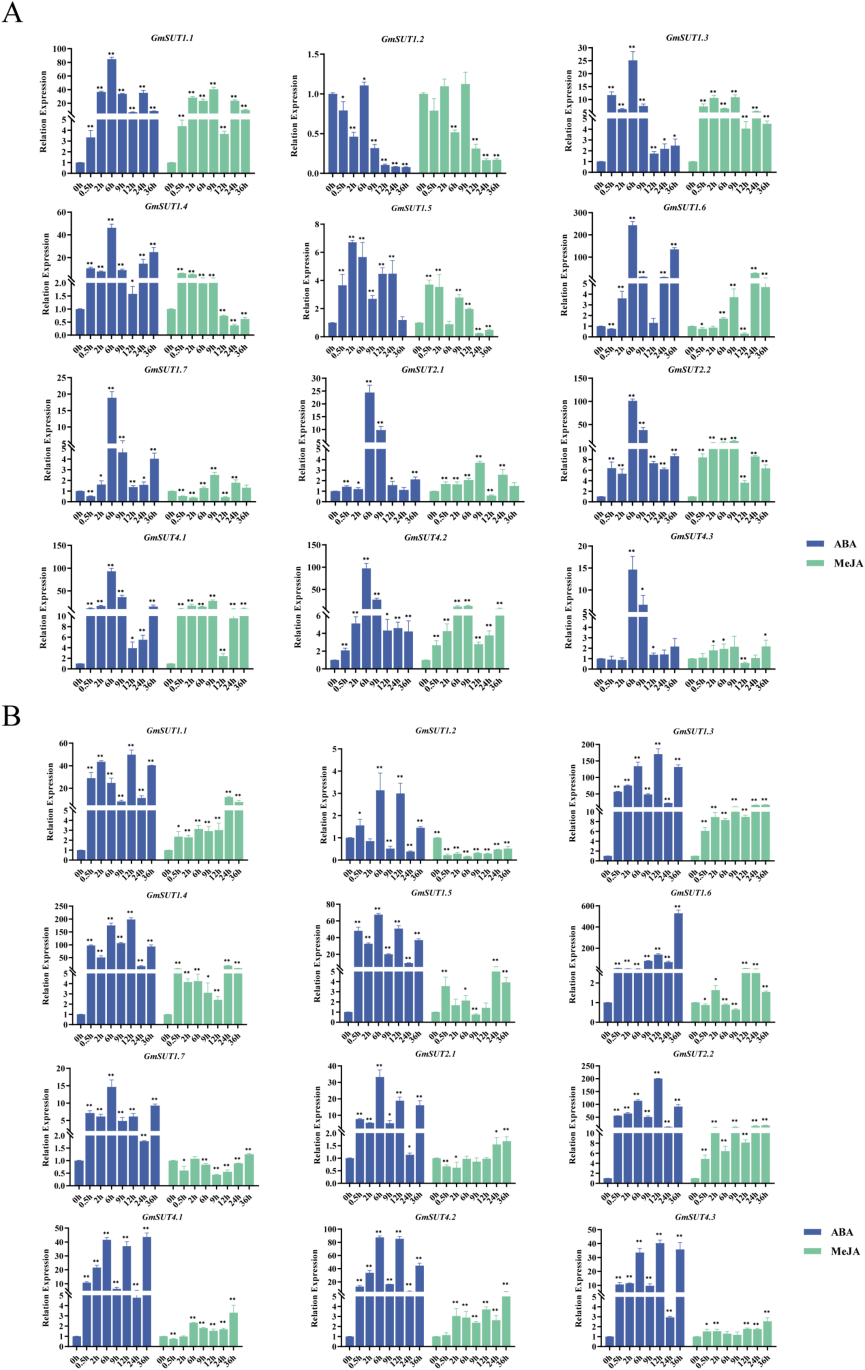
Expression analysis of 12 *GmSUT* genes following ABA and MeJA treatments by qRT-PCR. **(A)** Relative expression of *GmSUT*s in roots. **(B)** Relative expression of *GmSUT*s in shoots. The y- and x-axes indicate relative expression levels and time after treatment, respectively. Statistical significance was assessed using a paired Student’s t-test. Values are means ± SD from three biological replicates with three technical replicates each. Asterisks indicate significant differences compared with the control (*P ≤ 0.05; **P ≤ 0.01).

Under ABA treatment in roots (**Figure 9A**), 11 out of 12 *GmSUT* genes were upregulated, with *GmSUT1.2* s being the sole member showing a downward trend. All upregulated genes peaked at 6 h post-treatment. Notably, *GmSUT1.1*, *GmSUT1.6*, *GmSUT2.2*, and *GmSUT4.2* exhibited pronounced upregulation, with expression levels exceeding 50-fold compared to the control, whereas *GmSUT1.2* was consistently and significantly downregulated. In shoots, ABA treatment induced a similar expression tendency (**Figure 9B**): 11 genes were upregulated, and only GmSUT1.2 was significantly repressed. However, expression peaks in shoots were concentrated between 12 h and 36 h, with GmSUT1.3, GmSUT1.4, and GmSUT1.6 showing the most substantial induction, exceeding 100-fold relative to the control.

Following MeJA treatment in roots, all examined *GmSUT* genes displayed differential expression: 9 of 12 were upregulated—a pattern similar to that under ABA—whereas*GmSUT1.2* and *GmSUT1.7* were downregulated, and *GmSUT4.3* showed no significant change. Unlike the response to ABA, no synchronized expression peak was observed across gene members under MeJA treatment. In shoots, MeJA also elicited differential expression across all *GmSUT* genes: *GmSUT1.2* and *GmSUT1.7* were downregulated, while *GmSUT1.6*, *GmSUT2.1*, and *GmSUT4.1* exhibited a “decrease-then-increase” expression pattern.

### GO enrichment, protein interaction and co-expression network analysis of *GmSUT*s

**Figure 10:**
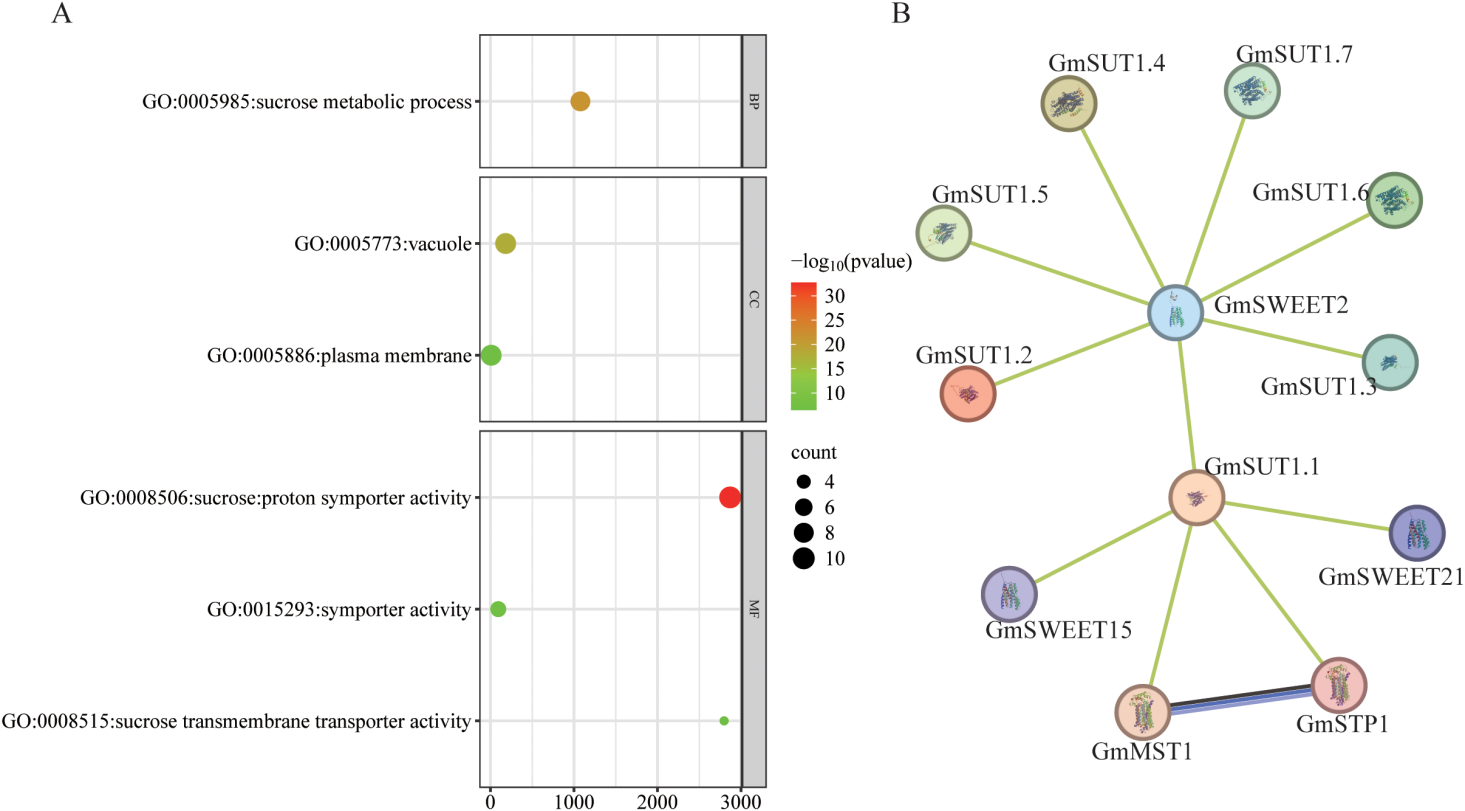
Gene Ontology (GO) enrichment analysis and the Protein-protein interaction network of the *GmSUT* genes. **(A)** Significant terms related to sucrose transport (GO:0015770), metabolic processes (GO:0005985), and transmembrane transporter activity (GO:0008515). Cellular localization predominantly involves vacuoles (GO:0005773) and plasma membranes (GO:0005886). **(B)** Protein-protein interaction network predicted by the STRING database. All protein–protein interaction and co-expression relationships presented here are computational predictions and should be interpreted as hypotheses rather than experimentally validated interactions.

Gene Ontology (GO) enrichment analysis of the 12 GmSUT members revealed highly significant enrichment in sucrose-related biological processes and molecular functions (**Figure 10A**). The most enriched terms included sucrose transport (GO:0015770, 8 genes, P = 4.94×10^-27^), sucrose metabolic process (GO:0005985, 8 genes, P = 2.80×10^-22^), and sucrose transmembrane transporter activity (GO:0008515, 3 genes, P = 3.10×10^-7^). Cellular component analysis showed strong localization to the vacuole (GO:0005773, 9 genes, P = 2.76×10^-18^) and plasma membrane (GO:0005886, 9 genes, P = 1.51×10^-7^), with molecular functions dominated by sucrose: proton symporter activity (GO:0008506, 10 genes, P = 1.86^-33^), consistent with their annotated roles in sucrose transport. Protein–protein interaction prediction via the STRING database suggested that GmSUTI subfamily members exhibit extensive interaction networks, including predicted associations with multiple sugar transporters such as *GmSWEET2*, *GmSWEET15*, *GmSWEET21*, *GmMST1*, and *GmSTP1* (**Figure 10B**).

Co-expression network analysis (**Supplementary Figure S2**) revealed the interaction pattern of *GmSUT*s: *GmSUT1.1/1.2/1.3* (SUTI) formed a core co-expression “triangle”, *GmSUT2.1/2.2* (SUTIIa) were directly connected, and *GmSUT4.2* (SUTIV) had an independent network, reflecting intra-subfamily synergy and inter-subfamily differentiation. Functional annotation showed *GmSUT4.2*’s network enriched ADP-ribosylation factor GTPase-activating protein-related genes, while SUTI/SUTIIa’s networks included transporters (e.g., nitrate transporter) and membrane proteins (e.g., MBOAT)—consistent with previous *GmSUT* subcellular localization (plasma membrane/cytoplasm). Additionally, SUTI’s network contained phloem proteins and cell necrosis control proteins, further supporting *GmSUT*s’ role in soybean abiotic stress adaptation via regulating sucrose transport and stress-related pathways.

## Discussion

Based on the soybean sugar transporter inventory reported previously (Guo et al., 2024), we integrated phylogenetic/structural evidence with expression profiling across development, abiotic stress, and phytohormone treatments to contextualize potential functional diversification of *GmSUT*s. Most *GmSUT*s cluster with homologs from *Arabidopsis thaliana*, *Glycine soja*, and *Medicago truncatula*, and the family retains an overall conserved structural framework (**Figure 2**). Together with their chromosomal distribution and duplication patterns (**Figures 3**, **4**; **Supplementary Table S5**), these features are most consistent with expansion via genome-duplication retention under functional constraint rather than extensive protein-coding innovation, implying that functional differentiation among *GmSUT* members may be driven largely by regulatory divergence. This rationale motivated our subsequent analyses of tissue/developmental specificity and stress- and hormone-responsive transcriptional reprogramming.

This pattern is consistent with the evolutionary stability of *SUT* gene structures reported across plant lineages (Sun et al., 2023; Fan et al., 2024; Hou et al., 2024), suggesting overlapping functions among members within a subfamily alongside specialization between subfamilies. Motif analysis further indicates that *GmSUT4.1* may represent a structurally atypical member, although it retains a conserved *SUT* domain. Importantly, phylogenetic analysis showed that *GmSUT4.1* clusters with *GmSUT4.3* within the SUTIV clade rather than forming an isolated branch. In addition, *GmSUT4.1* retains a conserved MFS-2 domain and displays detectable transcriptional responses under multiple stress and hormone treatments. These features support its classification as a non-canonical, lineage-specific member of the *SUT* family in soybean. By contrast, most SUTI members harbor eight or more motifs, indicating a more complete structural framework and a potentially broader functional scope. Collectively, these structural differences may underlie subfamily-specific transport properties while preserving core *SUT* functions in soybean.

The conserved architectures yet divergent subfamily features of *GmSUT*s raise the expectation of functional specialization, which is supported by their pronounced tissue- and developmental stage-dependent expression patterns. In particular, *GmSUT1.2* and *GmSUT1.3* showed consistently high expression in R1-stage flowers and remained prominent in R4-stage leaves, which is consistent with the established importance of SUT/SUC transporters reported in other species (Santiago et al., 2020; Li et al., 2022; Bavnhøj et al., 2023; Koyamatsu et al., 2023). The enrichment of expression in R1 flowers (**Figure 7B**) suggests that these genes may prioritize sucrose supply during flowering and early pod formation, a stage critical for yield establishment. Such cross-variety consistency further indicates that key *GmSUT* members maintain conserved roles in developmental carbon partitioning. Notably, promoter analysis (**Figure 6**) revealed abundant hormone- and stress-responsive cis-elements across the family, with ABREs present in 83% of *GmSUT* promoters, MeJA-responsive CGTCA/TGACG motifs in 75%, and low-temperature responsive elements (LTR) in 42%. Although motif presence alone does not demonstrate transcriptional regulation, this cis-element landscape provides a plausible regulatory context for the observed expression divergence and is consistent with promoter-level features reported for sucrose transporter gene family in other species (Ibraheem et al., 2010). Together, these observations motivated us to further examine how *GmSUT* transcription responds under abiotic stress conditions and hormone treatments.

Based on the developmental and tissue-specific expression patterns, the stress-responsive profiles of *GmSUT* genes suggest that soybean rapidly reconfigures sucrose transport capacity to support carbon redistribution under adverse conditions. The overall inducibility of most family members—together with coordinated responses within SUTIIa and SUTIV—fits the broadly conserved view that SUT transporters contribute to stress adaptation by sustaining source–sink fluxes when growth and metabolism are challenged, as documented in Arabidopsis and Solanaceae species (Gong et al., 2015; Chen et al., 2022) and in rice where *OsSUT1* is dynamically regulated under salt and drought to maintain photosynthate transport (Siahpoosh et al., 2012; Zhao et al., 2022). Importantly, the concurrent presence of gene- and tissue-specific repression in soybean (e.g., persistent suppression of *GmSUT1.2* in shoots under salt/drought and inhibition of *GmSUT1.5* in roots under cold stress) argues against a purely “global upregulation” model and instead supports a fine-tuning strategy in which selected transport steps are constrained to prevent inefficient carbon export or to prioritize stress-mitigation demands. This selective downregulation may reflect regulatory specialization of particular paralogs, making *GmSUT1.2* and *GmSUT1.5* promising candidates for subsequent functional studies. Given the tight crosstalk between phytohormone signaling and abiotic-stress responses, we next evaluated ABA and MeJA-associated expression to infer potential upstream regulatory cues shaping these transcriptional modes.

Phytohormone-responsive expression of *GmSUT* genes provides mechanistic clues for how soybean coordinates sucrose transport with stress signaling. The broadly inducible ABA pattern—contrasted by the persistent repression of *GmSUT1.2*—suggests that soybean *SUT* transcription is tightly embedded in ABA-centered programs that prioritize carbon reallocation during stress. This interpretation aligns with evidence from Arabidopsis showing that specific SUC/SUT members contribute to abiotic stress tolerance through ABA-dependent pathways and can modulate sucrose distribution and ABA accumulation (Gong et al., 2015; Jia et al., 2015). Notably, the tissue-dependent timing of ABA responsiveness in soybean further implies that ABA may couple source–sink adjustments with organ-specific needs (e.g., early root demand versus later shoot recovery), providing a framework to interpret the root–shoot phase shift observed here. In contrast, the more heterogeneous MeJA-associated response—lacking a single synchronized family-wide program—points to greater regulatory plasticity, consistent with the idea that hormone-related circuits can differentially wire sucrose transporter regulation to balance carbon allocation with growth–defense trade-offs under fluctuating environments (Liang et al., 2023). Together, these hormone-layer patterns support a model in which ABA acts as a dominant integrator of stress-driven sucrose partitioning, whereas JA-related signaling refines this allocation in a gene- and tissue-specific manner. In addition to ABA, hormone control of source–sink partitioning can involve other pathways such as auxin-regulated carbohydrate allocation (Zhao et al., 2022). More broadly, *SUT* expression and phloem loading are responsive to environmental and metabolic cues that help maintain carbon balance under changing conditions (Xu et al., 2018). In soybean, these pathways likely converge to shape tissue-specific *GmSUT* dynamics, providing a regulatory framework to interpret the coordinated transcriptional shifts observed under stress and hormone treatments and the functional modules inferred below.

GO enrichment highlights terms associated with sucrose transport and proton-coupled transmembrane activity, reflecting the conserved annotation and subcellular localization characteristics of *SUT* family members (Hirose et al., 1997; Fan et al., 2023). Consistent with this functional context, interaction predictions place SUTI members within broader sugar-transport modules, including associations with SWEET and other transporter families. Notably, the predicted linkage to *GmSWEET2* is of interest because *GmSWEET2* is a sugar transporter reported to influence soybean seed traits (Wang et al., 2024); given that *SUT*s are likewise sugar transporters, this predicted co-module association is intriguing and may point to coordinated regulation among distinct transporter families. Similar cooperation among transporters with different affinities and localization has been shown to influence transport output in other systems (Reinders et al., 2002), supporting the plausibility of combinatorial regulation in soybean. More broadly, the inferred network-level integration of sugar transporter families with stress- and hormone-related regulation is consistent with observations across plant lineages (Cao et al., 2019; Zhang et al., 2021; Ma et al., 2024). Importantly, these interaction/co-expression relationships should be interpreted as computational predictions that motivate further mechanistic testing, rather than evidence of direct physical interactions.

Overall, these results indicate that soybean *GmSUT* genes are evolutionarily conserved yet transcriptionally diversified, and that their coordinated regulation under development, abiotic stress, and phytohormone cues likely underpins dynamic sucrose partitioning in soybean.

## Conclusion

This study delivers a thorough genomic and functional analysis of the *SUT* gene family in *Glycine max*. Twelve non-redundant *GmSUT* genes were found and sorted phylogenetically into three subfamilies—SUTI, SUTIIa, SUTIV. The key findings are as follows. In terms of structural conservation, conserved motifs and domains (GPH-sucrose superfamily) were evenly distributed among subfamilies, except for the atypical *GmSUT4.1*. Regarding stress-responsive regulation, cis-element analysis detected abundant hormone-and stress-related elements (e.g., ABRE in 83% of promoters and LTR in 42%), which was consistent with the qRT-PCR data. The data show that multiple *GmSUT* genes were notably induced under ABA, MeJA, salt, drought, cold, and alkali treatments. From an evolutionary perspective, collinearity analysis revealed that *Glycine max* has a closer evolutionary relationship with *Glycine soja* than with *Arabidopsis thaliana* or *Medicago truncatula*. This analysis also supports the lineage-specific expansion of the SUTI subfamily on Chr16. All these results highlight the key part *GmSUT* genes play in mediating sucrose transport and enabling stress adaptation. The identified genes, particularly those that are persistently upregulated under abiotic stresses, are seen as promising candidates for genetic engineering to boost soybean resilience. Future research should center on functionally verifying individual *GmSUT* members in stress signaling pathways.

## Materials and methods

### Plant materials, growth conditions and stress treatments

The seeds of soybean (*Glycine max* cv. Williams 82) were provided by the Soybean Germplasm Bank of the National Center for Soybean Improvement, Nanjing Agricultural University.

Soybean plants (*Glycine max* cv. Williams 82) were grown in pots using vermiculite. Under a 14 h light/10 h dark photoperiod, seedlings were cultivated in a greenhouse at 26°C and 70% humidity. For subsequent experiments, plant materials were grown for approximately 14 days until the development of trifoliate leaves before treatment initiation.

For hormone stress treatments, Williams 82 seedlings were hydroponically treated with 100 µM ABA or 100 µM MeJA solutions. Shoot and root tissues were sampled at seven time points (0.5, 2, 6, 9, 12, 24, and 36 h) post-treatment. For salt and alkali stress treatments, seedlings were hydroponically exposed to 100 mM NaCl or 100 mM NaHCO3 solution, respectively. All shoot and root tissues were harvested at 0.5, 2, 6, 9, 12, 24, and 36 h after each treatment. For low-temperature treatment, seedlings were maintained in a growth chamber at 4°C, and tissues were sampled at seven time points (0.5, 2, 6, 9, 12, 24, and 36 h) post-treatment. For drought stress, seedlings were removed from vermiculite and subjected to rapid dehydration in a growth chamber, with tissues collected at 0.5, 2, 6, 9, 12, 24, and 36 h after treatment. Untreated seedlings served as controls. After each treatment, leaf samples were immediately frozen in liquid nitrogen and stored at -80°C for total RNA extraction. Roots were collected as root samples, and stems plus leaves were pooled as shoot samples. Three biological replicates were used for each treatment, each with three technical replicates.

### Sequence acquisition and gene identification

To study the relationship and classification of *SUT* family members in *Glycine max*, an evolutionary tree was constructed with 53 protein sequences including 9 AtSUCs, 5 OsSUTs, 4 ZmSUTs, 7 MtSUTs, 16 GsSUTs, and 12 GmSUTs. Protein sequences, CDS sequences, and annotation files for *Glycine max*, *Glycine soja*, *Oryza sativa*, *Zea mays*, *Arabidopsis thaliana*, *Medicago truncatula* were downloaded from the Phytozome database.

Specifically, protein sequences of *Arabidopsis thaliana* sucrose transporters (AtSUCs) were retrieved from the TAIR10 database and used as queries for BLASTP searches against the Glycine max Wm82.a4.v1 protein database using TBtools, resulting in 24 candidate proteins. In parallel, Hidden Markov Model (HMM) searches were conducted using two conserved domains characteristic of the SUC/SUT family, MFS-1 (PF07690) and MFS-2 (PF13347), obtained from the Pfam database. These HMM searches identified 522 proteins containing the PF07690 domain and 50 proteins containing the PF13347 domain in the soybean genome.

To ensure both sequence similarity to known SUC proteins and appropriate conserved domain architecture, candidate genes identified by BLASTP were further filtered by intersection with HMM search results. Conserved domains in the retained proteins were subsequently confirmed using the Phytozome database. Redundant transcripts derived from the same genomic locus were removed by retaining a single representative isoform, resulting in a final set of 12 non-redundant GmSUT genes.

Protein physicochemical properties, including amino acid length, molecular weight, and isoelectric point (pI), were calculated using the ExPASy server. Gene structure and chromosomal localization were determined based on the GFF3 annotation file of Glycine max using TBtools. Subcellular localization of GmSUT proteins was predicted using the WoLF PSORT online (https://wolfpsort.hgc.jp/). These predictions are used as supportive information and should be interpreted cautiously, as they are not experimentally validated.

### Multiple sequence alignment, phylogenetic analysis and collinearity analysis

Multiple sequence alignments were performed using ClustalX 2.11 with default parameters, based on *SUT* protein sequences from *Glycine max, Glycine soja, Oryza sativa, Zea mays, Arabidopsis thaliana, Medicago truncatula*. Following alignment, phylogenetic analysis was conducted using the Maximum Likelihood (ML) method in MEGA software (version 11 or later), with 100 bootstrap replicates to assess node reliability (Thompson, 1997; Tamura et al., 2021).

Collinearity analysis was performed using TBtools to identify syntenic gene pairs within the Glycine max genome and between G. max and other species. We used TBtools to detect collinearity blocks by comparing gene orders and identifying syntenic gene pairs. The identified collinearity pairs were highlighted in the generated synteny plot to visually emphasize the gene relationships. The final figure was modified and annotated within TBtools to improve clarity and highlight the syntenic relationships.

### Motif prediction, gene structure analysis and Cis-regulatory elements analysis

To investigate the conserved motifs of soybean *SUT* proteins, the identified *SUT* protein sequences were submitted to the MEME (Multiple EM for Motif Elicitation) program for motif discovery. The maximum number of motifs was set to 10, with other parameters retained as defaults. The GFF3 annotation file of *Glycine max* was downloaded from the Phytozome database, and exon/intron positional information for *SUT* genes was extracted from this file. Gene structure diagrams were generated using TBtools. The GFF3 file and genome sequence were used to extract a 2 kb sequence upstream of the start codon of the *GmSUT* gene, which was then submitted to the PlantCARE website for cis-element analysis and identification.

### Acquisition and processing of *GmSUT* gene expression data, GO enrichment analysis, and protein–protein interaction prediction

The expression levels of *GmSUT* genes across different soybean varieties were retrieved from the SoyMD database (https://yanglab.hzau.edu.cn/SoyMD/#/; Yang et al., 2024). Analyses focused on extracting and summarizing *GmSUT* gene expression values across the selected varieties, following the data processing standards established in SoyMD. Protein sequences were uploaded to STRING (https://cn.string-db.org/), perform the analysis, and download the image. DAVID was used to (https://davidbioinformatics.nih.gov/) database, the functions of *GmSUT* members were enriched, and visualization was conducted using R.

### Quantitative real-time PCR validation

To validate the RNA-seq results, quantitative real-time PCR (qRT-PCR) was performed to determine the relative expression levels of *GmSUT* genes in treated samples. Gene-specific primers were designed using Primer Premier 5 and are listed in **Supplementary Table S7** (**Supplementary Material**). The soybean gene *GmActin11* (*Glyma.18G290800*) was used as the internal reference gene, and relative expression levels were calculated using the 2^-ΔΔCt^ method (Livak and Schmittgen, 2001). Three independent biological replicates were included for each treatment, and each biological replicate was analyzed with technical replicates. All primers showed single peaks in melting-curve analysis, indicating specificity.

## Data Availability

The original contributions presented in the study are included in the article/**Supplementary Material**. Further inquiries can be directed to the corresponding author/s. The genome sequences of *A. thaliana*, rice, maize, alfalfa, soybean and wild soybean were downloaded from Phytozome database (https://phytozome-next.jgi.doe.gov/).
