# Supplementary figures and images for: Genome-wide analysis and functional characterization of the SUT gene family associated with stress tolerance in Glycine max
Source: Front Plant Sci. 2026 Feb 11;17:1717720. doi: 10.3389/fpls.2026.1717720 (PMC12932439; doi:10.3389/fpls.2026.1717720)

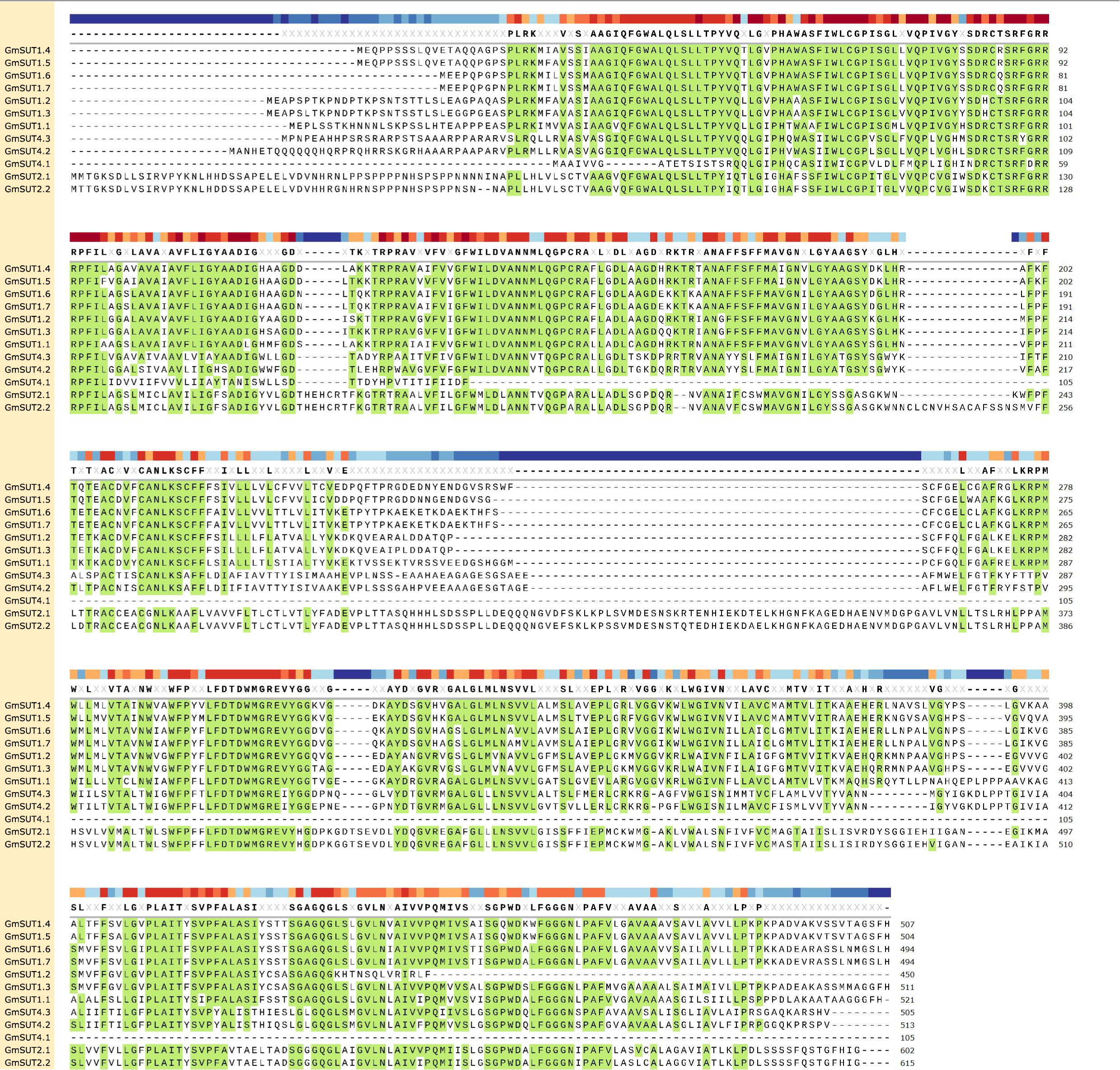

Supplement: Supplementary file 1 [file Image1.tif]

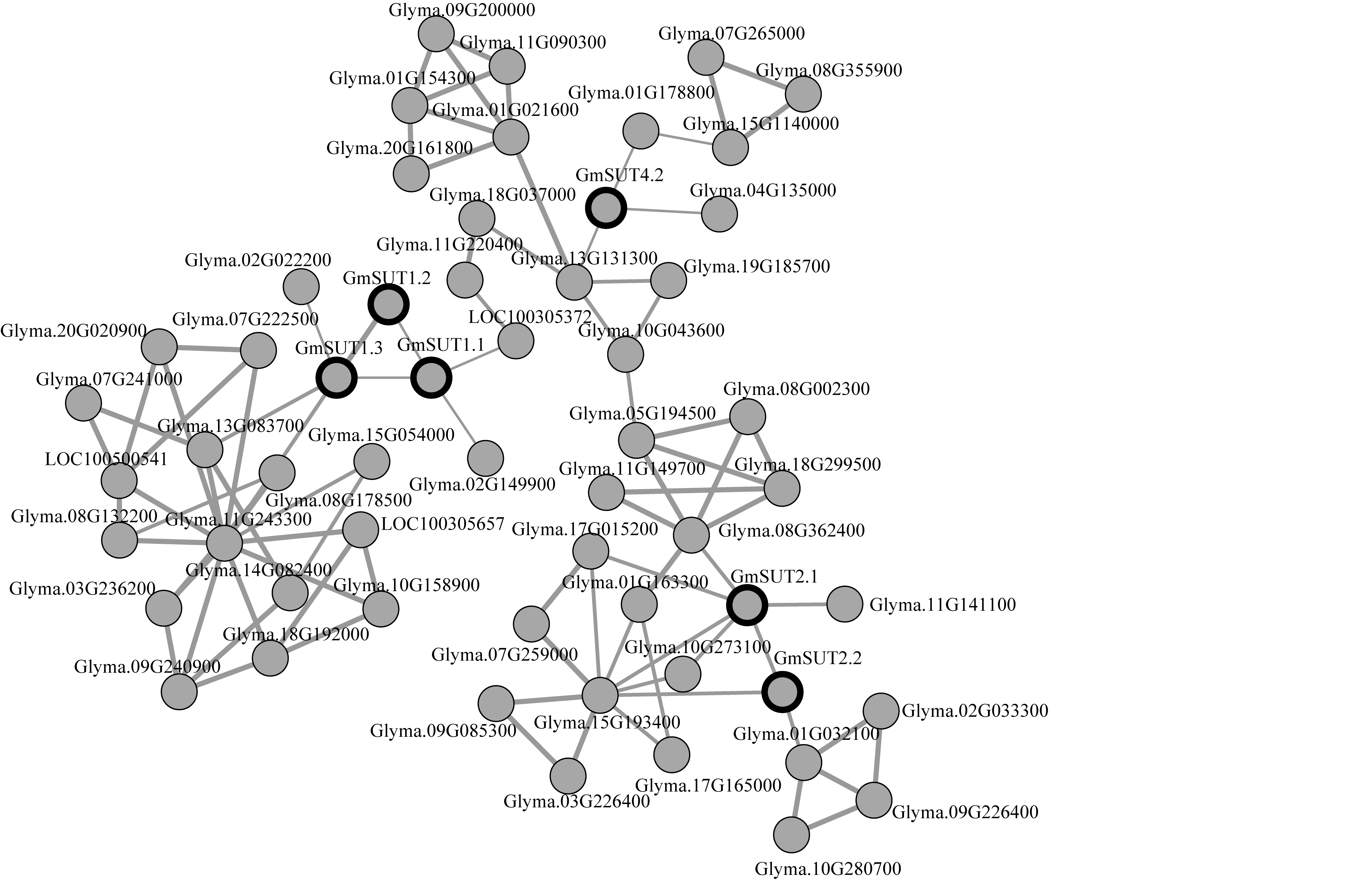

Supplement: Supplementary file 2 [file Image2.tif]
